# Factors associated with distress over time in women with breast cancer undergoing radiotherapy: insights from a pilot study assessing a digital information tool

**DOI:** 10.1007/s00520-025-09798-8

**Published:** 2025-08-12

**Authors:** Annika Grynne, Sofi Fristedt, Désirée Bourghardt Wiklund, Frida Smith, Maria Browall

**Affiliations:** 1 Department of Nursing, School of Health and Welfare, Jönköping University, Jönköping, Sweden; 2 School of Research, School of Health and Welfare, Jönköping University, Jönköping, Sweden; 3 Institute of Gerontology, School Health and Welfare, Jönköping University, Jönköping, Sweden; 4 Department of Health Sciences, Lund University, Lund, Sweden; 5 Breast and Melanoma Surgery Section, Sahlgrenska University Hospital, Gothenburg, Sweden; 6 Regional Cancer Centre West, Gothenburg, Sweden; 7 Department of Technology Management and Economics, Chalmers University of Technology, Gothenburg, Sweden; 8 Faculty of Caring Science, Work Life and Social Welfare, Department of Caring Sciences, University of Borås, Borås, Sweden; 9 Department of Oncology, Institute of Clinical Sciences, Sahlgrenska Academy, University of Gothenburg, Gothenburg, Sweden

**Keywords:** Distress, Digital health, Health literacy, Digital health literacy, Self-efficacy, Breast cancer, Radiotherapy, Virtual reality

## Abstract

**Purpose:**

A cancer diagnosis and treatment pose significant physical and psychological challenges. The study aimed to explore factors associated with distress over time in women diagnosed with breast cancer undergoing radiotherapy (RT) with access to a digital information tool, specifically examining if factors such as health literacy and self-efficacy had any influence on distress.

**Methods:**

In this pilot randomised controlled trial, women were assigned to an intervention group (*n* = 59) with access to a digital information tool or a control group (*n* = 52). Assessments were conducted at baseline, one week before RT (FU1), one week post- (FU2), and six months after treatment (FU3). Distress was measured at all time points, and associated factors were evaluated at baseline and six months.

**Results:**

In the intervention group, a statistically significant reduction in distress was observed over time (FU1, *p* = .009; FU2, *p* < .001; FU3, *p* < .001). The control group showed a significant reduction at FU3 (*p* = .009). Quade’s ANCOVA revealed no significant differences between the groups in distress prevalence (*F* = 3.460, *p* = .066). No significant changes in health literacy or self-efficacy were observed over time.

**Conclusion:**

The results indicate no statistically significant effect on distress; however, there is a potential indication of a reduction in distress, suggesting that the digital information tool may offer some benefits. Further research is required to confirm this relationship.

## Background

Breast cancer is the most common cancer among women worldwide, with 8,837 women diagnosed in Sweden in 2023 [1, 2]. Treatment often requires patients to make critical health-related decisions [3]. This necessitates the provision of extensive health information about the diagnosis and treatment options, which patients are expected to understand and adhere to. However, stressful situations, such as receiving a diagnosis and being scheduled for treatment, and situations perceived as threatening, can significantly impact both the individual’s emotional state and their ability to comprehend the information provided [4, 5]. As a result, patients often retain only some parts of the information shared with them, leading to increased distress [6, 7].

A breast cancer diagnosis and its treatment frequently cause high levels of distress [4]. A study using the National Comprehensive Cancer Network (NCCN) Distress Thermometer scale (range 0–10, cut-off ≥ 4) reported a mean score of 2.96 (± 1.85) among newly diagnosed breast cancer patients, with 43.71% (*n* = 73/167) scoring 4 or higher, indicating high distress. Distress encompasses a range of negative psychological and physical challenges that can significantly impact how patients perceive and manage their cancer treatment [8]. Breast cancer treatment often involves multiple, complex therapies, with radiotherapy (RT) being a common approach. RT improves local tumour control and increases overall survival rates [9]. However, the high-tech RT environment and associated treatments are often unfamiliar to non-professionals, potentially eliciting fear and distress [10]. Many patients report unmet needs for support and information and research findings present high prevalence of distress before, during, and after RT [6, 11, 12]. In a multicentre cohort of 1042 cancer patients undergoing RT, nearly two-thirds reported increased distress (cut-off ≥ 5) [13]. Another study found that 48% of breast cancer patients experienced increased distress in anticipation of RT [14].

Health literacy is a crucial factor in understanding health information. Health literacy is defined as the knowledge, motivation, and competencies that determine a person’s ability to access, understand, evaluate, and apply health information to promote and maintain good health and make appropriate decision-making [15, 16]. A commonly used definition of health literacy ranges from basic skills in reading (functional health literacy) to more complex skills in evaluating health information and communication (critical and communicative health literacy) [16]. Factors such as age (≥ 65 years) and marital status have been associated with health literacy challenges among cancer patients [13, 17, 18]. Health literacy is recognised as dynamic, a process that evolves over time and varies depending on social determinants, emotional factors, and individual characteristics [16, 19]. Limited health literacy can affect patients’ comprehension of health information and their ability to ask questions during the in-person clinical meetings [3, 20]. Similarly, digital health literacy, the ability to obtain, comprehend, process, and communicate health information using digital health technologies, is becoming increasingly important in healthcare [21].

Applying user-friendly digital information technology that has been co-designed with end users fosters the development of a person-centred, health literacy-friendly environment [22, 23]. These environments include policies, health information, and services within the health system, all of which influence how people access, understand, appraise, and apply health information and use services [22–24]. Similar to health literacy, high self-efficacy is crucial for women who are adjusting to breast cancer diagnosis and treatment [25, 26]. Self-efficacy is typically defined as the belief in one’s ability to successfully perform behaviours necessary to manage new and stressful situations [26]. In cancer care, self-efficacy has been linked to an individual’s comprehension of health information, ability to manage treatment side effects, and the maintenance of daily activities [25]. The use of digital health in oncological care has proven to have multiple benefits, including the facilitation of accessible, and understandable, evidence-based information [27]. These benefits do not only align closely with the principles of person-centred care but also have the potential to enhance individuals’ perceived informational support, thereby increasing their self-efficacy [27, 28]. Furthermore, research suggests that digital health can enhance knowledge acquisition and develop health literacy [29, 30].

For this study, digital health refers to information and communication technology that works to advance population wellness and improve health care and health outcomes [31]. To date, the number of studies having explored the potential of digital health by comparing an app-based digital information tool with traditional methods (oral and written) for delivering health information in the context of radiotherapy-related distress among women diagnosed with breast cancer is sparse [32, 33].

The study aimed to explore factors associated with distress over time in women diagnosed with breast cancer undergoing radiotherapy with access to a digital information tool. The following research questions will be addressed:

1. Are there differences in levels of distress between the intervention and control groups at baseline and over time?
2. Are health literacy and self-efficacy associated with distress in the overall group and within subgroups (intervention and control) at baseline and over time?
3. What demographic and clinical factors are associated with distress in the overall group and within subgroups (intervention and control) at baseline and over time?

## Methods

This manuscript reports on a pilot randomised controlled trial (RCT). The project received ethical approval from the Swedish Ethical Review Authority (Dnr 2020–00170).

### Trial design and study setting

**Fig. 1:**
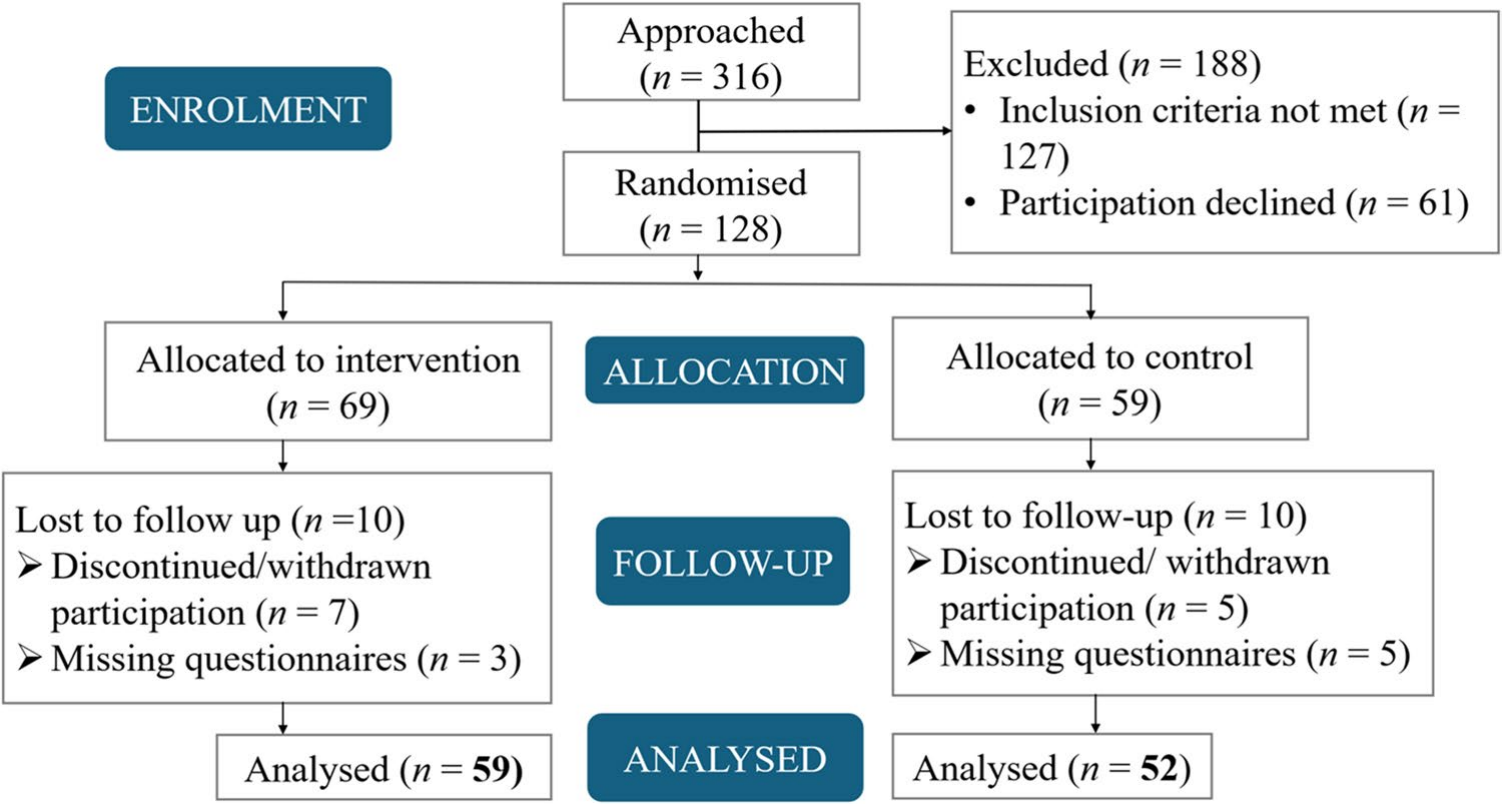
CONSORT flow diagram of the phases of a randomised trial of two groups (that is, enrolment, intervention allocation, follow-up, and data analysis enrolment process)

This study employed a multi-centre, non-blinded, 2-arm design involving participants diagnosed with breast cancer and scheduled for post-operative RT. Initially, a full-scale, prospective, longitudinal RCT was planned to take place at two hospital sites (a university hospital and a regional hospital) in Western Sweden [34]. The trial was prospectively registered in the ClinicalTrials.gov database (Registration number: NCT04394325), and the initial protocol was published as an open-access RCT study protocol [34]. However, the COVID-19 pandemic imposed significant restrictions on research activities. Research nurses were redeployed to support routine hospital care, causing recruitment for the study to be paused for an extended period. These challenges, combined with the need to ensure relevance to emerging technological advancements, led to the reclassifying of this study as a pilot RCT. The ambition was to evaluate the feasibility of conducting a full-scale RCT in the future. Participant enrolment and follow-up data are presented in the Consolidated Standards of Reporting Trials (CONSORT) flow diagram Fig. 1.

### Procedures

Recruitment began in September 2020 and was completed by April 2022. The data collection commenced in September 2020 and was completed by November 2022. Women attending a routine clinical visit following breast cancer surgery were invited to participate in the study. After the clinical consultation, the first author or a research nurse assessed each patient’s interest in and eligibility for participation. Eligibility criteria included the following: Diagnosis of non-metastatic breast cancer, allowance for neo-adjuvant and endocrine therapies, age 18 years or older, scheduled hospital RT preparation visit no earlier than one day after inclusion, fluency in Swedish, access to a smartphone. Patients with a history of prior RT treatment were excluded from the study. Eligible patients provided oral informed consent. In accordance with the Declaration of Helsinki, participants received both oral and written explanations of the study’s objectives, design, content, the researchers’ access to medical records, randomisation process, and the voluntary nature of their participation [35].

### Randomisation

Participants were randomly assigned (1:1) to the intervention or control group using permuted block randomisation with blocks of 2 based on an a priori randomisation list. Randomisation took place following screening and after obtaining the participants’ consent. Each participants allocation was concealed from the study investigators until assignment to either of the two groups. All participants received standard care and health information (both oral and written), regardless of group allocation. Participants in the intervention group were granted access to the digital information tool. Research staff assisted with downloading the apps onto the participant’s smartphone or tablet, demonstrated the use of the virtual reality headset, and provided both oral and written instructions for app navigation. Intervention group participants were encouraged to continue using the tool at home while awaiting RT and throughout their treatment period.

### Digital information tool — the Digi-Do

Digi-Do is an innovative, interactive digital health tool utilising virtual reality technology. It was co-designed by researchers, an innovation design team, healthcare professionals, and patients undergoing RT, as described in a previous study [36]. The tool comprises two mobile applications that can be rapidly adapted and iterated in real-time to meet user needs.

#### First app: virtual reality simulation

The first app uses virtual reality technology to simulate a visit to the RT department, allowing users to familiarise themselves with the clinical environment before their first visit. For users who prefer a non-immersive experience, navigation can be performed using a finger on the mobile screen. The app enables users to control their experience by exploring various areas of the department, including high-technology treatment rooms, waiting rooms, and restrooms. A voice-over provides explanations of the user’s location and the clinical processes they encounter.

#### Second app: health information

The second app presents health information related to cancer and RT in three formats: evidence-based flashcards, animated videos, and practical information. This approach allows users to access information in a way that best suits their personal needs.

#### User feedback

Our previous qualitative evaluation found Digi-Do to be user-friendly, offering accessible, evidence-based health information while allowing users to explore the clinical environment before their initial visit [18]. Participants valued the ability to share Digi-Do with family and friends, which enhanced their sense of preparedness and understanding. Offering users the flexibility to choose the type and amount of information with which they engage aligns closely with a person-centred approach [28]. Digi-Do was made freely available for download in Sweden via existing app stores for Android and iOS to ensure broad accessibility without developing a new digital platform. To prevent control group participants from accessing the tool, the app’s name was not disclosed prior to randomisation. To our knowledge, no participants in the control group accessed Digi-Do during the study period.

### Data collection

#### Overview

Data were collected through self-reported questionnaires administered at four time points: baseline, one week before the start of RT (FU1), one week after RT (FU2), and six-month post-completion of RT treatment (FU3). Baseline represents the time point when participants had received information from the surgeon and registered nurse regarding the results of the operation and were informed that the next stage would involve RT. They were also notified that the oncology department would contact them with further information. Determining whether the participants met the criteria for inclusion in the study and the randomisation process was also conducted at this stage. Additionally, the intervention group received the Digi-Do tool. Baseline data included demographic variables (e.g., age, cohabitation status) and clinical factors (e.g., tumour-specific details). Distress was measured at all four time points, while other outcomes, i.e., health literacy, digital health literacy and self-efficacy, were evaluated at baseline and six months after RT using five validated questionnaires. The CONSORT flow diagram Fig. 1 presents the final sample of 111 participants included in the analysis, out of 316 individuals initially approached. The primary reasons for declining participation were related to life circumstances, including personal or family responsibilities. Discontinuation from the study was mainly attributed to participants feeling exhausted or lacking the energy to complete the questionnaires, often due to unforeseen personal circumstances, ongoing treatment, or general fatigue. Additionally, some participants were excluded due to non-return of completed questionnaires.

#### Distress Thermometer

Distress was measured using the NCCN Distress Thermometer, a validated self-assessment tool designed to evaluate distress levels over the preceding week, including the current day [8]. Participants rated their distress on an 11-point scale ranging from 0 (no distress) to 10 (extreme distress), with scores of ≥ 4 indicating high distress [37]. The Distress Thermometer also includes a checklist covering 39 items across five domains: practical, family, emotional, physical, and spiritual/religious concerns. However, this checklist was not analysed in the current study. The Swedish version of the tool, which has demonstrated strong reliability (Cronbach’s alpha > 0.70), was employed [38].

#### Functional health literacy

Functional health literacy (FHL) evaluates basic reading and comprehension skills required for understanding health information in daily life [39]. To better align with the digital health context, the Swedish FHL scale was modified for this study. One item assessing recall of health information was added, while two items less relevant to digital health information access were removed. The final scale comprised three items rated on a 5-point Likert scale (1 = never to 5 = always). Psychometric testing of the FHL scale in a Swedish setting demonstrated strong reliability, with a Cronbach’s alpha value of 0.86 [40].

#### Communicative and critical health literacy

The communicative and critical health literacy (C&C HL) questionnaire evaluates the advanced communication and critical analysis skills needed to derive meaning from and apply health information in various contexts [41]. The Swedish C&C HL scale consists of five items rated on a 5-point Likert scale (1 = strongly disagree to 5 = strongly agree), with higher scores indicating greater health literacy [41]. The scale includes items related to advanced social and cognitive skills in communication and the application and appraisal of various health information sources. Cronbach’s alpha for the Swedish C&C HL was 0.87 [40]. Both FHL and C&C HL were manually categorised into three levels (sufficient, problematic or inadequate) based on established guidelines [39, 40]. Additionally, both scales were dichotomised into “sufficient” vs “limited” (including problematic/inadequate) [40].

#### Digital health literacy

Digital health literacy was assessed using the Swedish version of the electronic Health Literacy Questionnaire (eHLQ) [42]. This tool measures an individual’s ability to engage with digital health technologies and has been validated across multiple clinical settings, demonstrating robust psychometric properties (Cronbach’s alpha > 0.7). The eHLQ comprises 35 items organised into seven domains: (1) ‘Using technology to provide health information’, (2) ‘Understanding health concepts and language’, (3) ‘Ability to engage with digital services’, (4) ‘Feeling safe and in control’, (5) ‘Motivation to engage with digital services’, (6) ‘Access to functional digital services’, and (7) ‘Digital services that fit individual needs’. Each item is rated on a 4-point ordinal scale (1 = strongly disagree to 4 = strongly agree), with domain-specific average scores calculated for analysis [42, 43].

#### General Self-Efficacy

The General Self-Efficacy (GSE) scale was used to measure participants’ confidence in their ability to manage life challenges. The GSE scale consists of 10 items rated on a 4-point Likert scale (1 = not at all true to 4 = exactly true). Higher score indicates high self-efficacy. GSE scores were dichotomised into low self-efficacy (≤ 30) and high self-efficacy (≥ 31) [44]. The Swedish version of the scale has demonstrated high internal consistency, with a Cronbach’s alpha of 0.91 [45].

### Statistical analysis

Data on baseline demographic and clinical factors are presented as the mean value for continuous variables and as proportions with percentages for categorical data. Differences between intervention and control groups were analysed using the Chi-square test and Fisher’s exact test for categorical variables and Student’s *t*-test for continuous variables.

Non-parametric tests were used to assess within-group changes using the Wilcoxon signed-rank test and between-group changes using the Mann–Whitney test, across time points (baseline – FU1, baseline – FU2 and baseline – FU3) in distress, health literacy, and self-efficacy. The median value for digital health literacy, eHLQ seven domains were calculated and compared between intervention and control groups. Repeated measures were employed to estimate marginal means of distress over time. Quade’s Analysis of Covariance (ANCOVA) was applied to assess group differences in distress scores between baseline and FU3, adjusted for baseline values. Logistic regression was used to identify factors associated with the binary variable “low vs. high” distress (cut-off ≥ 4) at baseline and at FU3, adjusted for baseline distress levels. Relevant variables based on previous literature, such as demographic and clinical factors (e.g., tumour subtype, treatment duration) FHL, C&C HL, digital health literacy, GSE score, and were also investigated [46, 47]. Univariable logistic regressions were conducted first to identify factors, based on weak conditions for association (*p* ≤ 0.1) to be included in the multiple logistic regression models Tables 2 and 3. Logistic regression was initially performed for the entire group (including intervention and control groups), followed by subgroup analyses based on the intervention and control groups’ allocation.

## Results

The trial flow chart Fig. 1 illustrates the final sample of 111 participants included in the analysis. Demographic and clinical factors were comparable between the intervention and control groups, with no statistically significant differences observed, except for treatment duration (measured as the number of RT days) Table 1.

**Table 1:** Demographic and clinical factors of the intervention- (*n* = 59) and the control groups (*n* = 52) at baseline

	Intervention group (*n* = 59)	Control group (*n* = 52)	*p*
Mean age in years	60.2,	61.7,	0.422^a^
SD, range	10.7, 34–84	9.18, 37–82	
Marital status, *n* (%)			0.665^b^
Co-habiting	43 (73)	35 (67)	
Work situation *n* (%)			
Retired	23 (39)	19 (36)	0.945^b^
Residential area *n* (%)			0.882^b^
Within 50 km from the hospital	39 (66)	36 (69)	
Within 50–250 km from the hospital	20 (34)	16 (31)	
Co-morbidities *n* (%)			0.127^b^
1–2 conditions	19 (32)	23 (44)	
> 2 conditions	1 (2)	3 (6)	
Invasive cancer *n* (%)	51 (86)	41 (79)	0.439^b^
Stage of invasive cancer *n* (%)			1.000^c^
1 = < 20 mm	35 (59)	29 (56)	
2 = 21–50 mm	13 (22)	10 (19)	
3 = > 50 mm	1 (2)	1 (2)	
Missing	10	12	
Tumour characteristics *n* (%)			0.085^c^
Luminal	48 (81)	38 (73)	
HER2 +	4 (7)	0 (0)	
Triple negative	0 (0)	2 (4)	
In situ	7 (12)	11 (21)	
Missing	0 (0)	1 (2)	
Treatment duration, days *n* (%)			0.042^b^
5	25 (42)	33 (64)	
15	34 (58)	19 (36)	
RT combined with another Treatment *n* (%)			0.458^c^
RT only	13 (22)	15 (29)	
RT, endocrine therapy	34 (58)	32 (61)	
RT, chemotherapy and endocrine therapy	10 (17)	4 (8)	
RT, chemotherapy	2 (4)	1 (2)	
Functional health literacy *n* (%)			0.169^b^
Sufficient	40 (68)	27 (52)	
Limited*	19 (32)	25 (48)	
Communicative & Critical health literacy *n* (%)			0.963^b^
Sufficient	32 (54)	28 (54)	
Limited*	26 (46)	24 (46)	
General Self-Efficacy *n* (%)			0.257^c^
Low, < 30	24 (41)	27 (52)	
High, > 31	35 (59)	25 (42)	

^a^Student’s *t*-test

^b^Chi-Square test

^c^Fischer’s exact test

*Limited = problematic & inadequate

### Prevalence of distress over time

**Fig. 2:**
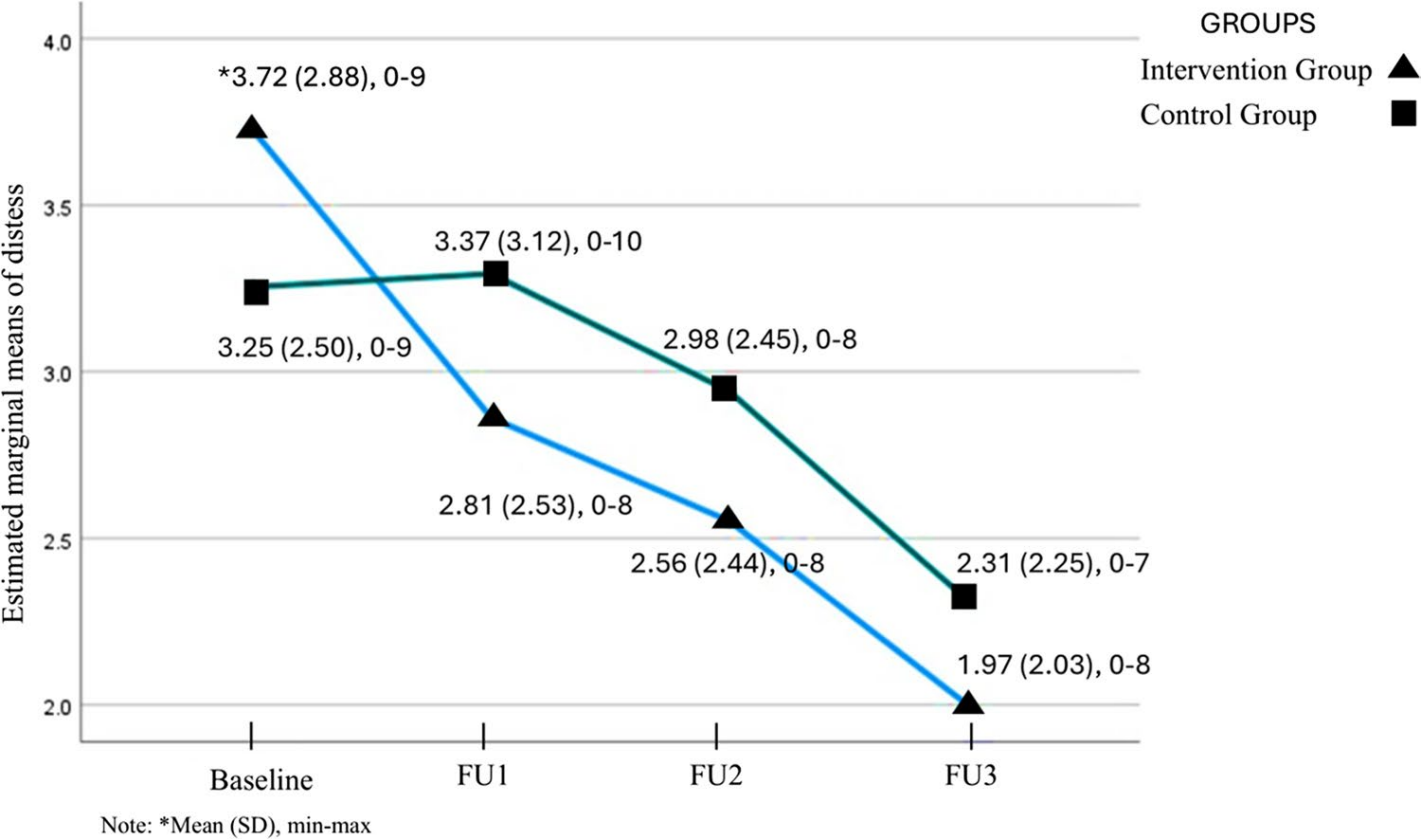
Estimated marginal means distress over time in the intervention- and control groups

Overall, the prevalence of distress reduced over time among the study participants, 44% reported high distress at baseline to 26% at FU3. At baseline, 49% (*n* = 29) of participants in the intervention group and 40% (*n* = 20) in the control group reported high distress levels. Figure 2 presents the self-reported distress scores over time (mean, SD). The non-parametric test Wilcoxon-Signed Rank test was used to assess distress over time in both groups. The intervention group exhibited a statistically significant reduction in distress scores from baseline to all follow-ups (FU1, *p* = 0.009; FU2, *p* = < 0.001; FU3, *p* = < 0.001). In contrast, the control group exhibited a significant reduction only between baseline and FU3 (*p* = 0.009), while changes at FU1 (*p* = 0.980) and FU2 (*p* = 0.209) were not significant. However, Quade’s ANCOVA analysis of between-group differences over time did not quite reach significant difference for distress prevalence between groups (*F* = 3.460, *p* = 0.066).

### Functional health literacy, communicative and critical health literacy and digital health literacy

At baseline, some individuals in both the intervention and control groups reported limited (problematic and inadequate) health literacy Table 1. No statistically significant changes were observed in Functional Health Literacy (FHL) or Communicative and Critical Health Literacy (C&C HL) over time, neither within nor between the groups. Analysis of the seven domains of the eHLQ revealed similar mean scores in both groups. However, a closer examination revealed some differences. In Domain 1: ‘Using technology to process health information’, the intervention group showed an increase in the median score from 2.86 at baseline to 3.00 at FU3 (indicating increased use of technology to process health information), while the control group’s median decreased from 3.00 to 2.80 (indicating less use of technology). In Domain 3: ‘Ability to actively engage with digital services’, the control group’s median score decreased from 3.20 to 3.00 (indicating reduced ability to engage), while no change was observed in the intervention group. Domain 4: ‘Feel safe and in control’ showed an increase in the median score in both groups, from 3.20 at baseline to 3.40 at FU3 (indicating participants felt safer and more in control). In the intervention group, a statistically significant change was found between baseline and FU3 in Domain 2: ‘Understanding health concepts and language’ (*p* = 0.037). Despite these observations, no statistically significant change was found between the groups in digital health literacy over time.

### General self-efficacy

Baseline measurements of GSE scores are presented in Table 1. At FU3, 64% (*n* = 38) of participants in the intervention group and 52% (*n* = 27) in the control group reported high levels of GSE (≥ 31). No statistically significant changes were observed over time within or between groups.

### Predictors of distress

#### Baseline factors associated with distress in the total sample

Univariable logistic regression of the entire participant group (intervention and control) identified factors for inclusion in the multiple logistic regression models: older age, vocational status (not retired, i.e., individuals still in the workforce), longer RT treatment, i.e., 15 days, low FHL, low self-efficacy, and digital health literacy (eHLQ) specifically domain 2, reflecting poor understanding of health concepts and language. Vocational status (OR 0.152, 95% CI [0.039 to 0.6], *p* = 0.007), and low self-efficacy (OR 0.908, 95% CI [0.827 to 0.998], *p* = 0.044) was significantly associated with increased distress at baseline. No other covariates showed statistically significant associations.

#### Factors associated with distress at follow-up 3 in the full sample

Factors included in the multivariable analyses to identify covariates associated with increased distress six months after RT completion (FU3) were tumour subtype (invasive tumour types, i.e., luminal, HER2 +, triple-negative vs. Ductal Carcinoma In Situ, DCIS), RT combined with another treatment (neoadjuvant/adjuvant chemotherapy and/or endocrine therapy vs. RT alone), low FHL, low self-efficacy, and eHLQ domains 2 (poor understanding of health concepts), 4 (feeling less safe and in control), and 6 (limited access to effective digital services). High distress at baseline was significantly associated with high distress at six months post-RT (OR 6.227, 95% CI [2.041 to 18.995], *p* = 0.001). None of the additional covariates demonstrated statistical significance.

#### Factors associated with distress in intervention and control groups

Subgroup analyses identified variables uniquely associated with distress in the intervention and control groups at baseline and six months post-RT (FU3). For the intervention group, the factors included in the multiple analyses were vocational status, invasive tumour subtype, longer RT treatment (i.e., 15 days), RT combined with another treatment rather than RT alone, and low self-efficacy. In the control group, variables included vocational status, worsening tumour grade (< 20 mm, 21–50 mm, > 50 mm), and digital health literacy in eHLQ domains 2 (poor understanding of health concepts and language), 6 (limited access to digital services that work), and 7 (limited access to digital services that suit individual needs).

Multivariable analyses for covariables associated with high distress at baseline revealed that none of the covariates significantly contributed to the model for the intervention group Table 2. Distress at baseline was significantly associated with increased distress at six months in both the intervention and control groups. Additional factors linked to increased distress at FU3 in the intervention group included low self-efficacy (*p* = 0.02), digital health literacy eHLQ, “less understanding for health concept and language” (domain 2) (*p* = 0.04), and “feeling less safe and in control” (domain 4) (*p* = 0.044). In the control group, RT combined with another treatment (*p* = 0.045) significantly contributed to the model. The results of the multiple logistic regression for both groups are presented in Table 3.

**Table 2:** Multiple logistic regression for subgroup (intervention and control) at baseline

		Dependent variable: low vs high distress								
		Intervention group (*n* = 59)					Control group (*n* = 52)			
		Exp(B)	95%	CI	*p*		Exp(B)	95%	CI	*p*
Covariate	Subgroup		Lower	Upper				Lower	Upper	
Vocational status		0.174	0.03	1.07	0.06		0.08	0.01	1.213	0.07
Tumour subtype	0 = DCIS, 1–3 Invasive	0	0		0.999		0.081	0.005	1.213	0.069
RT treatment in days	0 = 5 1 = 15	1.113	0.92	1.348	0.27		0.6	0	9E + 13	0.976
Tumour grade	< 20 mm, 21–50 mm, > 50 mm	1.635	0.271	9.882	0.592		0.868	0.676	1.116	0.27
RT combined with another treatment	0 = RT only, 1–2 chemotherapy, endocrine therapy	0.791	0.166	3.777	0.769		31.783	1.577	640.5	0.024
General Self-efficacy, Range 10–40	< 30 low, > 31 high	0.849	0.7	1.029	0.095		43.142	1.454	1280.1	0.03
Domain 2: ‘Understanding of health concepts and language’	1 = poor 4 = comprehensive	0.466	0.077	2.818	0.406		1.128	0.881	1.444	0.341
Domain 6 ‘Access to digital services that work’	1 = poor 4 = comprehensive	5.421	0.227	129.59	0.297		34.353	0.653	1806	0.08
Domain 7 ‘Access to digital services that suit individual need’	1 = poor 4 = comprehensive	0.342	0.022	5.332	0.444		0.001	0	0.412	0.025
Constant		1E + 11			0.999		0.291	0.011	7.492	0.456

**Table 3:** Multiple logistic regression for subgroup (intervention and control) at FU3

		Dependent variable: low vs high distress*												
		Intervention group (*n* = 59)					Control group (*n* = 52)							
		Exp(B)	95%	CI	*p*		Exp(B)	95%		CI		*p*		
Covariate	Subgroup		Lower	Upper				Lower		Upper				
Distress at baseline	low vs high	11.35	1.58	81.47	0.016		5.108	1.037		25.15		0.045		
Age in years	34 to 84 years	0.938	0.853	1.031	0.185		0.965	0.887		1.049		0.397		
RT combined with another treatment	0 = RT only, 1–2 chemotherapy, endocrine therapy	0.47	0.097	2.283	0.349		0.275	0.078		0.972		0.045		
Functional Health Literacy	1 = sufficient, 2 = problematic, 3 = inadequate	3.967	0.743	21.18	0.107		0.83	0.173		3.993		0.817		
General Self-efficacy Range 10–40	< 30 low GSE, > 31 high GSE	12.56	1.499	105.3	0.02		0.934	0.806		1.083		0.365		
Domain 2 ‘Understanding of health concepts and language’	1 = poor 4 = comprehensive	0.198	0.042	0.928	0.04		0.201	0.025		1.604		0.13		
Domain 4 ‘Feel safe and in control’	1 = poor 4 = comprehensive	0.772	0.6	0.993	0.044		2.343	0.403		13.62		0.343		
Constant		136.8			0.366									

*Adjusted for baseline values distress

## Discussion

This pilot RCT served as a first step toward a full-scale trial, focusing on the feasibility of the study design rather than evaluating the full effect of the intervention [48]. Although no statistically significant intervention effect was observed, the study contributes to the growing body of literature exploring the potential of digital health tools in supportive cancer care. A high prevalence of distress was evident in both the intervention and control groups, consistent with existing evidence suggesting elevated distress among women undergoing RT for breast cancer [33, 49]. In the intervention group, which had access to Digi-Do, the reduction in distress levels was statistically significant across all follow-ups (FU1–FU3), whereas in the control group, a significant reduction was only observed at FU3. However, it is important to acknowledge that statistical significance is more easily attained when reductions occur from a higher baseline level of distress, particularly when interpreting percentage changes or relative differences. This reflects established statistical principles, as highlighted by Greenland [50], and underscores the complexity of evaluating subjective outcomes such as distress. Factors such as the timing of assessment, individual variation (e.g., disease trajectory), and context-specific influences all contribute to the challenge of reliably capturing changes in such a multifaceted and personally experienced phenomenon.

When considering the findings on distress it is important to consider the care trajectories of the participants. Despite the absence of data on time since diagnosis was not collected, baseline measures were taken approximately one-month post-surgery. Some participants had also received neoadjuvant chemotherapy, which may have either increased distress due to cumulative treatment burden or decreased it through earlier adaptation. Notably, baseline distress was higher in the intervention group compared to the control group. While this difference may have been coincidental, it is also possible that group allocation influenced participants’ self-reported responses, given that randomisation occurred prior to baseline assessments. For some, being assigned to the intervention group may have inadvertently heightened distress, particularly among individuals with limited digital health literacy, anxiety around technology use, or uncertainty about the tool. Conversely, those in the control group may have experienced disappointment at not receiving the intervention, which could have similarly influenced their responses. These considerations underscore the importance of collecting baseline data prior to randomisation in future trials to minimise allocation-related bias and ensure more accurate group comparisons.

In the control group, distress levels increased one week prior to radiotherapy (FU1), followed by a general decline in both groups over time. This initial elevation may be indicative of anticipatory anxiety associated with the forthcoming treatment. Previous qualitative findings suggest that Digi-Do helped participants familiarise themselves with the RT setting, potentially mitigating distress [18]. Systematic reviews support the value of digital health interventions, including virtual reality, in reducing distress [32, 47]. Interestingly, while many interventions use immersive experiences, our qualitative findings showed a preference for non-immersive, mobile-based navigation [18], an aspect worth exploring in future studies.

Although the between-group comparison did not reach statistical significance (*p* = 0.066), this may be due to the limited sample size, data variability, or the potential lack of sensitivity in the measurement tools used [51]. Nevertheless, participants in the intervention group reported increased confidence in using technology to manage health information (eHLQ Domain 1), highlighting the potential of digital health tools to promote person-centred, health literacy-friendly environments [16, 52]. Digi-Do may enhance patients’ sense of control and facilitate more effective communication with healthcare professionals, thereby fostering more active engagement in their care [18].

The literature reveals inconsistent findings regarding predictors of distress among women with breast cancer. However, vocational status and low self-efficacy were associated with higher baseline distress in our sample [25, 33]. Moreover, elevated distress at baseline was significantly associated with higher distress six months post-treatment, reinforcing the value of early screening and tailored support. A person-centred approach, acknowledging patients’ narratives alongside tools like the Distress Thermometer, can help identify needs and introduce timely interventions, such as digital information tools, for support [47, 52].

The relatively short follow-up period may partly explain the absence of statistically significant findings. Another possible explanation is that constructs such as health literacy and self-efficacy are not easily influenced over a limited timeframe. Although both are dynamic, they typically evolve gradually through accumulated experiences and ongoing support [17, 25]. Nevertheless, they remain important to consider, as they may moderate how individuals perceive and respond to distress. Importantly, while immediate improvements may not be visible, supporting health literacy and self-efficacy remains essential, as they contribute to individuals’ capacity to engage with care, process health-related information, and cope with emotional challenges. Digital health tools, when thoughtfully designed, may provide a foundation for such support by offering tailored, accessible information that patients can engage with on their own terms. In this context, Jaensson and colleagues [53] emphasise the value of incorporating qualitative data to better understand dimensions of patient experience that may be overlooked by quantitative measures alone. Capturing such experiences is essential for uncovering nuanced insights into how individuals engage with and benefit from interventions, thereby enhancing understanding of their clinical relevance and informing the development of more responsive and supportive digital health solutions.

While the study’s prospective design, repeated measures, and inclusion of a control group represent key strengths, limitations must be acknowledged. The requirement for fluency in Swedish may have excluded vulnerable populations, thereby limiting the generalisability of the findings. Moreover, educational attainment and household income, factors known to be associated with health literacy [3], were not collected, representing a missed opportunity for more nuanced analysis. Additionally, a considerable number of individuals declined participation due to treatment-related anxiety, potentially leading to an underrepresentation of those experiencing higher levels of distress. These factors highlight the need for cautious interpretation of the results and underscore the importance of further research with larger, more diverse samples to validate and extend these findings.

## Conclusion

Although no statistically significant intervention effect on distress was identified, this pilot RCT provides valuable insights into the feasibility of implementing a digital information tool within the context of RT for breast cancer. The observed reduction in distress over time in the intervention group suggests that information tools such as Digi-Do may usefully complement clinical care by enhancing patients’ understanding and support a health literacy-friendly environment underpinned by person-centred care principles. By enabling individuals to access information at their own pace and according to their personal needs, Digi-Do may facilitate informed preparation ahead of clinical encounters. In turn, this allows healthcare professionals to tailor their communication and provide targeted, person-centred care and support based on each patient’s expressed information needs.

### Clinical implications and future research recommendations

The findings from this pilot study provide valuable insights that can inform the design of future RCTs evaluating the efficacy of digital information tools within RT settings. Despite the absence of statistically significant intervention effects on distress, the results highlight the potential of digital information tools like Digi-Do to support patient understanding and readiness for treatment. Recognising the limited understanding of the multidimensional factors contributing to distress during RT, further research is warranted to explore how such interventions may best address the diverse and evolving needs of patients. Mixed methods research offers a promising approach to achieving a more comprehensive understanding of the role digital health can play in supportive cancer care, something that cannot be fully captured through singular, quantitative approaches alone. As distress is a complex, multifactorial, and inherently subjective phenomenon, it may not always be adequately assessed through standardised instruments. Exploratory approaches, particularly those incorporating qualitative inquiry, may yield deeper insights into individual patient experiences, thereby enhancing the clinical relevance of intervention outcomes [54]. Understanding how individuals respond to RT, shaped by personal experiences, cognitive evaluations, and levels of comprehension, may help identify subgroups at higher risk for increased distress. Future research should aim to explore these variations, identifying potential mechanisms or moderators that influence distress trajectories. Such knowledge could support the development of more tailored and equitable oncological care pathways.

Given the complexity of distress within the cancer trajectory, even modest improvements may carry meaningful clinical value. To fully assess the impact and relevance of such changes, it is essential to move beyond statistical significance alone. As emphasised by Jaensson and colleagues [53], capturing patient experiences through qualitative inquiry is critical for revealing insights that quantitative measures may fail to detect. These approaches are particularly well-suited to illuminating the nuanced, subjective aspects of distress and patient support needs, thereby contributing to a stronger and more person-centred care evidence base for the clinical utility of digital health interventions in supportive cancer care.

## Data Availability

Data is available upon request from the main author.
